# Recalcitrant eumycetoma of the foot: Financial burden a major barrier on the road of recovery

**DOI:** 10.1371/journal.pntd.0008356

**Published:** 2020-08-06

**Authors:** Gagandeep Singh, Reshu Agarwal, Swati Khullar, Immaculata Xess, Vinod Kumar Sharma, Ankur Goyal

**Affiliations:** 1 Department of Microbiology, All India Institute of Medical Sciences, New Delhi, India; 2 Department of Dermatology and Venereology, All India Institute of Medical Sciences, New Delhi, India; 3 Department of Radiodiagnosis, All India Institute of Medical Sciences, New Delhi, India; Johns Hopkins Bloomberg School of Public Health, UNITED STATES

## Summary

Eumycotic mycetoma has been recently included in the World Health Organization’s list of neglected tropical diseases. It is the first fungal infection to get such significant attention by the international health community. The disease is distributed worldwide, with majority of cases reported from tropical and subtropical countries like India, Mexico and Sub-Saharan areas of Africa. However, due to chronic course of disease or lack of adequate medical services, the reported cases represent only the tip of iceberg. In addition to this, even after diagnosing the disease on time, successful outcomes are further hampered by various factors like duration, cost, toxicity, and compliance to treatment. We report a case of refractory eumycetoma who initially responded to the therapy but interruption due to financial constraints led to worsening of lesions and, finally, ending into amputation.

A 30-year-old male resident of Rajasthan, India, presented to our Dermatology outpatient department with complaint of recurrent swelling on the left sole with multiple discharging sinuses. A small papule developed 15 years back, which slowly increased in size associated with bloody discharge and black granules. The patient could not recall any history of trauma. Due to recurrent episodes, multiple surgical excisions were performed providing temporarily relief only. He was then referred to our hospital, which is the apex health care institute of India. Grains collected from discharging sinuses were 1 mm to 2 mm, hard in consistency, crushed, and subjected to microscopy and culture. The potassium hydroxide (KOH) mount of discharge with black grains did not reveal any fungal elements. However, the KOH-calcofluor staining showed the presence of septate hyphae, and *Madurella* sp. grew on culture. Despite on itraconazole and terbinafine therapy, recurrence occurred after 5 months, with repeat culture also growing *Madurella* sp. Surgical excision was done, and posaconazole was added along with continuation of terbinafine. The patient showed clinical improvement after eleven months of treatment, after which he stopped treatment due to financial constraints. The lesions recurred within the following months, and he finally had to undergo amputation.

Management of recurrent cases with newer potent antifungal, such as posaconazole, should be considered early in the course of the disease. Financial burden, prolonged duration, tolerability, and compliance with medical therapy are issues which need to be addressed. Such patient and their kin should be counseled for completing the regimen in spite of high cost of the drugs as it might prevent amputation, which in turn might affect their livelihood.

## Introduction

Mycetoma is a serious, mutilating, chronic inflammation of cutaneous and subcutaneous tissue following traumatic inoculation of filamentous fungi (eumycetoma) or filamentous bacilli (actinomycetoma). It is characterized by clinical triad of tumefaction, multiple draining sinuses, and discharge of granules. The true incidence of disease is unknown with majority of cases reported from tropical and subtropical countries, especially sub-Saharan areas of Africa, India, and Mexico [1,2]. The disease is listed among neglected tropical disease by the World Health Organization (WHO) in 2016 [3]. Treatment depends upon the causative agents and extent of disease. Management of these patients is often challenging and usually requires prolonged drug therapy with or without surgical intervention. Recurrences are common, and compliance to treatment further complicates the prognosis [4]. We report a case of refractory eumycetoma who initially responded to the therapy, but interruption due to financial constraints led to worsening of lesions and finally ended in amputation.

## Case report

A 30-year-old male resident from Rajasthan, India, and worker in a company, presented to the Dermatology outpatient department (OPD) of our hospital with recurrent episodes of swelling over left middle sole with multiple discharging sinuses. He was apparently alright 15 years ago when he developed a small papule that slowly grew in size with occasional discharge of pus and black granules. No history of trauma and other associated risk factors were recalled by the patient. Due to recurrent episodes, multiple surgical excisions were performed at the local hospital, providing temporary relief only. In the last 2 years, there was an increase in the swelling, which made walking more difficult. The local physician started itraconazole, 100 mg twice daily. This regimen was continued for two years without much improvement in the lesions. Thereafter, he was referred to a tertiary care center for further management.

**Fig 1:**
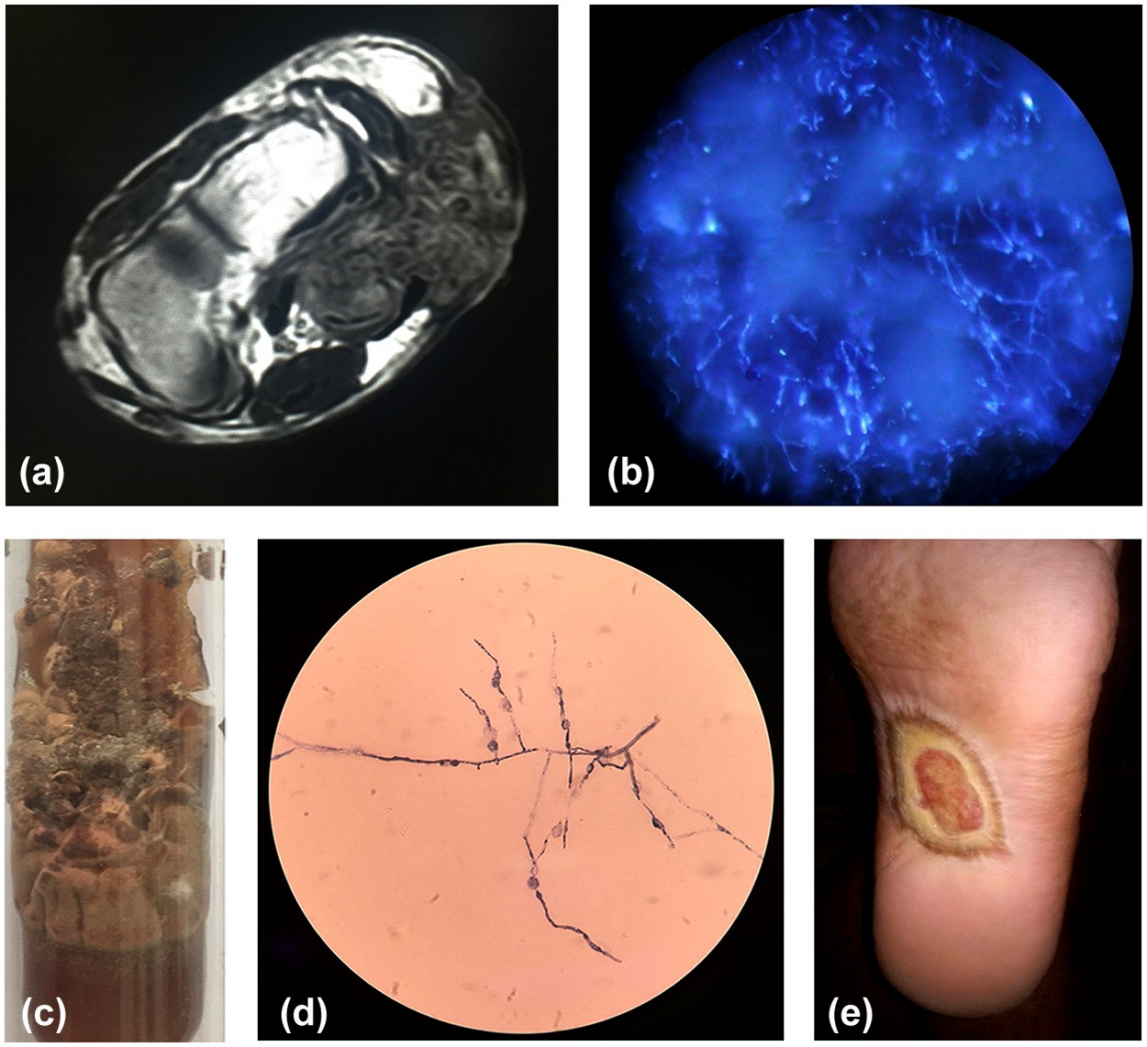
Clinical features and diagnostic findings in the present case with eumycetoma. **(A)** MRI (T2) of the left foot showing heterogeneously enhancing soft tissue with “Dot in Circle” sign (white arrow). (**B)** KOH**-**calcofluor white staining of the grain showing compact septate hyphae. (**C)** Growth on Sabouraud Dextrose Agar. **(D)** Lactophenol cotton blue mount of the microculture from the colony showing *Madurella* sp. **(E)** Lesion after surgical treatment.

Physical examination revealed a single, stellate shaped ulcer covering an area of 2 cm^2^ to 3 cm^2^ over the left middle sole. The lesion showed multiple pin head sized sinuses with discharging black granules. No other bony deformity or skin lesions were present. The MRI of the left foot showed soft tissue enhancing lesion with “dot in circle sign” suggestive of mycetoma (Fig 1A). Histopathological examination of biopsy taken from the lesion showed dense chronic inflammatory cells with granulation tissue along with occasional giant cells and pigmented septate hyphae.

Grains collected from discharging sinuses were 1 mm to 2 mm, hard in consistency, crushed, and subjected to microscopy and culture. On KOH-calcofluor white stain, compact granules showing multiple septate hyphae were observed ((Fig 1B) Modified Ziehl–Neelsen staining for aerobic actinomycetes including *Nocardia* sp. was negative. Culture of the granules on Sabouraud Dextrose Agar was velvety, brown, producing a brownish diffusible pigment (Fig 1C). Lactophenol cotton blue mount of the microculture shows dense, melanized mycelium along with phialides with minute conidia in short chains (Fig 1D). Based on colony morphology and microscopic findings, the isolate was identified as *Madurella* sp.

Surgical excision was performed, and the patient was started on itraconazole 200 mg BD and terbinafine 250 mg OD. Following mild improvement in the wound, he was discharged with advice to continue medication for six months (Fig 1E). On follow up at six months, the lesion did not show much improvement, and he was readmitted for the same. Microscopic examination of grains in 10% KOH-calcofluor white stain again showed septate hyphae, and culture on SDA with antibiotics again yielded *Madurella* sp. Surgical excision was performed, and the patient was shifted to syrup posaconazole 300 mg BD. The patient was discharged on a two-drug regimen, which included posaconazole 300 mg QID and terbinafine 250 mg OD. The patient came for follow up after eleven months. The lesions had improved dramatically and healed totally with extensive scarring. He was advised for continuation in therapy with regular follow-up every month. However, on observing significant improvement in the lesions, the patient stopped treatment thereafter. The lesions recurred after complete healing in the following month, and he had to undergo amputation of the foot in November 2018.

## Tutorial

### What is the population that is usually affected by eumycetoma?

Mycetoma is caused following a traumatic inoculation of the etiological agent. As such, the population strata most predisposed to the disease are the farmers, loggers, woodcutters, lumberjacks; owing to their nature of work, they are put most at risk in acquiring the disease. These daily wagers often are the sole earning member of the household with no surety of next day’s work.

### What are the challenges in early diagnosis of eumycetoma?

Since initially mycetoma presents as a painless swelling, most patients tend to ignore the disease symptoms to the extent possible so as to ensure their daily livelihood. This swelling slowly progresses to involve the skin, followed by deeper structures, including leading to bony destruction and deformity and then they show up at healthcare facilities, since the disease starts affecting their working capabilities. Every visit to the hospital means a loss of a day’s work and hence puts them at an economic setback. Mycetoma treatment is long term generally ranging from 6 months to many years and has a high recurrence rate.

### What is the economic impact of treating eumycetoma with currently available antifungal agents?

Treatment of mycetoma is long term, generally ranging from 6 months to many years and is associated with high recurrence rates. Apart from the surgical management, azoles including itraconazole and posaconazole are most often used for the treatment of eumycetoma [5,6]. Table 1 shows the dose of the various drugs used for the treatment and the cost incurred for treatment for 6 months. As per the Situation Assessment Survey of Agricultural Households (SAS) in 2013 (70th Round) [7], the average annual income of a farm house hold in India is 77,112 rupees. Even though posaconazole therapy has shown better outcomes, affected individuals with low average annual income are incapable of affording the treatment for even up to 6 months due to financial constraints. Cost factors also pose a hindrance for other treatment modalities in these patients. Ultimately, these patients succumb to the pressure of treatment costs and end up stopping the treatment, which further deteriorates the disease and adds to the economic burden in the form of disabilities.

It is painful to know that how a disease like mycetoma puts the patient and his family into a vicious cycle of debt, financial problems, and deranged health. In low socioeconomic countries with majority of population residing in the rural setting, this particular population strata often misses the basic requisites of living and can be seen working barefoot. It has been seen that most of the cases of mycetoma have been reported with foot involvement. Non adherence to treatment and follow-up due to financial constraints aggravates the disease process and can even lead to amputation of the diseased region. The sole earning member of the family becomes incapable to support his dependents. The economic burden of mycetoma is still unexplored, and what we discuss is still the tip of the iceberg. It is important to realize that a disease process like mycetoma, which can be a result of a trivial trauma, is not just a health problem but has social as well as economic connotations. Although prevention of this disease might not be a realistic dream at present, however. increasing awareness, exploring sensitive modalities for early diagnosis, initiating prompt treatment, and provision of drugs at cheaper and affordable price is the need of the hour.

**Table 1:** Average cost of a six-month course of the commonly used antifungal agents in the treatment of eumycetoma. (The average annual income of a farm house hold in India is 77,112 rupees).

Antifungal	Dose	Price (rupees)	Cost for 6 months of treatment in rupees	Approximate cost for 6 months of treatment in US$
Itraconazole	200–400 mg/day	59.96–190 per 100 mg	21,585–43,171	310–620
Posaconazole	800 mg/day	16,000 per 105 ml (40mg/ml)	576,000	8230
Terbinafine	500 mg/day	156–175 per 250 mg	56,160	802

### How can we ensure early diagnosis and initiation of appropriate treatment in cases of eumycetoma?

Firstly, the most important aspect of early diagnosis is sensitization of the population at risk. This brings the “public health” officials in the picture, in which there is an urgent need for them to conduct sessions at primary health centers and educate people regarding the symptoms, seeking early medical advice and initiation of therapy. They can also help by distribution of printed education material. Focal group discussion among local communities might also be beneficial. In addition, prevention of injury by encouraging use of closed shoes and wearing gloves can also be highlighted.

Secondly, strengthening our laboratories at the primary health care level is also extremely important. Direct microscopy can be of immense significance in establishing the correct diagnosis. It is important to differentiate actinomycetoma with eumycetoma, as the treatment is entirely different. However, using only 10% KOH needs some level of skill to report fungal elements. The addition of calcofluor white fluorescent stain increases the sensitivity to a great extent. In the present report, we could easily observe the compact fungal elements in the form of granules using calcofluor white, which were missed on 10% KOH mount.

### Ethics statement

This case is published with permission of the patient, and written informed consent has been obtained.

**Key learning points**

- Early diagnosis is the key to appropriate treatment of eumycetoma.
- Antifungal agents like posaconzole, when started early, can prevent morbidity.
- The approximate treatment cost for six months ranges from 20,000–600,000 rupees, depending upon the antifungal prescribed.
- Awareness among population at risk (e.g., farmers, laborers, carpenters, etc.) can make them seek medical advice early on.
- Public health department can play an important role in sensitization.
- Strengthening and upgradation of all public health laboratories to be able to make a diagnosis of fungal infection is warranted.
